# The impact of student computer competency on e‐learning outcomes: A path analysis model of virtual learning infrastructure, collaboration, and access to electronic facilities

**DOI:** 10.1002/nop2.2130

**Published:** 2024-03-14

**Authors:** Hamid Sharif‐Nia, Gökmen Arslan, Jonathan Reardon, Kelly‐Ann Allen, Lan Ma, Long She, Esmaeil Hoseinzadeh, Pardis Rahmatpour, Siavash Moradi, Fatemeh Khoshnavay Fomani, Nassim Ghahrani

**Affiliations:** ^1^ Education Development Center Mazandaran University of Medical Sciences Sari Iran; ^2^ Department of Nursing, Amol Faculty of Nursing and Midwifery Mazandaran University of Medical Sciences Sari Iran; ^3^ Department of Psychological Counseling Burdur Mehmet Akif Ersoy University Burdur Turkey; ^4^ Durham University Durham UK; ^5^ School of Educational Psychology and Counselling, Faculty of Education Monash University Clayton Victoria Australia; ^6^ Centre for Wellbeing Science, Faculty of Education University of Melbourne Parkville Victoria Australia; ^7^ Sunway Business School Sunway University Sunway City Selangor Malaysia; ^8^ Department of Nursing, Faculty of Medical Sciences, Gorgan Branch Islamic Azad University Gorgan Iran; ^9^ School of Nursing Alborz University of Medical Sciences Karaj Iran; ^10^ Community Medicine Specialist, Education Development Center Mazandaran University of Medical Sciences Sari Iran; ^11^ School of Nursing and Midwifery Tehran University of Medical Sciences Tehran Iran; ^12^ Mazandaran University of Medical Sciences Sari Iran

**Keywords:** accessibility, computer competency, e‐learning outcomes, infrastructure, student collaboration

## Abstract

**Aim:**

This study explored the influence of student computer competency on e‐learning outcomes among Iranian nursing students and examined its mediating role in the relationship between virtual learning infrastructure, student collaboration, access to electronic facilities, and e‐learning outcomes.

**Design:**

A cross sectional study.

**Method:**

A self‐administered online survey was used from August to October 2022, with a sample size of 417 nursing students selected through convenience sampling. Descriptive statistics, correlation analyses, and PROCESS macro v4.1 (Model 4) were used for data analysis.

**Results:**

The results revealed that virtual learning infrastructure, access to electronic facilities, and student collaboration, significantly predict student computer competency and e‐learning outcomes. Virtual learning infrastructure and access to electronic facilities were found to be the strongest predictors of student computer competency, while student collaboration had a smaller but still significant effect. Student computer competency was found to mediate the relationship between virtual learning infrastructure, access to electronic facilities, student collaboration, and e‐learning outcomes.

## 1 INTRODUCTION

The integration of the internet into higher education has led to significant changes, especially related to the growing acceptance of virtual education (Attardi & Rogers, 2015; Lakbala, 2016; Nazari et al., 2020). The COVID pandemic expedited this shift, with institutions pivoting to virtual learning to meet social distancing guidelines (She et al., 2021), thereby highlighting both the benefits and challenges of online education.

E‐learning provides convenient access to educational materials, overcoming geographical barriers and facilitating global student interaction (Akhter et al., 2021). Web‐based delivery allows assignments to be tackled from any location and at any time, offering flexible learning opportunities that cater to each student's unique needs. It also enables teachers to personalize course content to further enhance the learning experience (Laksana, 2022; Sharif Nia et al., 2023). Additionally, online learning has been linked to an increase in behavioural, affective, and cognitive student engagement, as well as increased academic self‐efficacy and student satisfaction (Sharif‐Nia et al., 2023; She et al., 2021).

Despite its many advantages, e‐learning is not without limitations. One major drawback is the potential lack of access to necessary technological infrastructure for economically marginalized students from lower socio‐economic areas, hindering participation in online learning (Malenya & Ohba, 2023). This can engender feelings of incompetence or anxiety, likely to adversely impact their academic performance and satisfaction (Absah et al., 2021). It is therefore imperative that governments and educational institutions implement efforts to ensure students facing educational barriers to receive adequate support, guaranteeing equitable access to quality education through e‐learning platforms (Moosavi et al., 2022). Furthermore, e‐learning in nursing students is recognized to have limitations, notably not offering the same level of interpersonal engagement, sense of community, or hands‐on experiences as traditional learning approaches (Innab et al., 2022; Luo et al., 2017). Recent research highlights the importance of student preparedness and computer proficiency for achieving optimal learning outcomes in online education (Rahmani & Nazemi Jenabi, 2020; Regmi & Jones, 2020). While virtual scenarios, games, and simulations are utilized in the electronic education of clinical nursing skills, it should be noted that these cannot fully and adequately substitute the learning of clinical skills for nursing students (Salmani et al., 2022).

While e‐learning provides students with faster and broader access to education content, it is important to recognize that a lack of learning support can have a detrimental effect. The shift to e‐learning presents significant challenges due to unequal access to technology and resources, especially in economically under‐resourced areas (Tate & Warschauer, 2022). Improving access to electronic facilities is crucial for ensuring equitable participation in modern education systems across all regions (Faja, 2013; Jahanbakhsh et al., 2021; Masoomifard, 2019). The inadequacy of educational infrastructure has led to discontent among students and staff, diminishing collaboration and cooperation in virtual environments.

## 2 LITERATURE REVIEW

### 2.1 Virtual learning infrastructure

Providing sufficient infrastructure to support information technology is crucial for launching and developing e‐learning platforms among nursing students (Faja, 2013; Jahanbakhsh et al., 2021; Masoomifard, 2019; Nazari et al., 2020). Unfortunately, some educational contexts, constrained by funding, lack the resources to support this (Laksana, 2022). Other studies have also highlighted the challenges posed by inadequate information technology infrastructure (Attardi & Rogers, 2015; Lakbala, 2016) and determined that, irrespective of the problem's root cause, it can obstruct the fundamental purpose of education: to support student learning (Moosavi et al., 2022). Research has also demonstrated that universities allocating funds to bolster electronic infrastructure typically observe better student outcomes (Rahmani & Nazemi Jenabi, 2020). This research highlights the crucial role information technology infrastructure plays in the success of e‐learning platforms. Recognizing the importance of virtual learning infrastructure to e‐learning outcomes prompts the first research question:

Question 1: Is there a significant relationship between virtual learning infrastructure and e‐learning outcomes?

### 2.2 Student collaboration

Placing student collaboration at the forefront of education is one of the facilitators of successful e‐learning (Regmi & Jones, 2020). Research indicates that nursing students particularly value the experience of cooperation and collaboration in e‐learning environments (Jahanbakhsh et al., 2021; Jowsey et al., 2020). Such collaborative efforts can amplify interactions with peers, lecturers, and the course content itself (Masoomifard, 2019), while also fostering a sense of community, pivotal for enhancing student engagement and motivation in e‐learning (Faja, 2013; Lee et al., 2019). However, the effective management of student collaboration warrants careful consideration. Therefore, exploring the relationship between nursing student collaboration and e‐learning outcomes can yield insights into cultivating productive collaboration in e‐learning environments. The importance of student collaboration prompts the second research question:

Question 2: Is there a significant relationship between student collaboration and e‐learning outcomes?

### 2.3 Access to electronic facilities

Access to electronic facilities is one of the most important factors for nursing student satisfaction with e‐learning (Mousavizadeh, 2022). While other factors, such as the instructor, course constituent interactions, and course management issues, are pivotal in achieving positive e‐learning outcomes and understanding satisfaction levels, access to electronic facilities stands out as a crucial factor (Nazari et al., 2020). Insufficient student access to resources has been associated with feelings of tiredness, fear, and anxiety (Dhawan, 2020; Tegowati et al., 2022) and increased access to electronic facilities enhances the potential for nursing students' learning, discussion and collaboration, and problem‐solving skills (Männistö et al., 2020). Moreover, recent studies reveal that the quality of electronic facilities, such as level of access, speed, and reliability, can significantly influence student engagement and motivation (Nambiar, 2020). This highlights the need for educational institutions to ensure not only access to electronic resources but also to uphold and enhance the quality of these resources to foster effective e‐learning. Therefore, the importance of accessibility in fostering student collaboration and promoting e‐learning prompts the third research question:

Question 3: Is there a significant relationship between student access and e‐learning outcomes?

### 2.4 Student computer competency

There is significant variability among nursing students with regard to computer competency and skills in general, and this impacts e‐learning acceptance and satisfaction (Hart, 2012; Nes et al., 2021). Students proficient in computer use tend to exhibit increased self‐confidence, self‐reliance, and independent learning, potentially finding e‐learning even more beneficial than traditional methods (Lin et al., 2007; Mosalanejad & Sobhanian, 2009). In essence, student success in e‐learning environments depends on possessing a set of prerequisite skills for e‐learning (Ojaghi et al., 2019). Mastering these skills and demonstrating competency in them can facilitate successful learning, foster creativity, and develop critical thinking skills and intellectual curiosity (Shuster & Pearl, 2011). However, it is important to acknowledge that not all students possess the requisite skills for success in e‐learning environments, potentially leading to frustration and disengagement (Al‐Maskari et al., 2022; Sathyan et al., 2022). Providing training and support for students lacking these skills can help to bridge the gap and ensure that all students have equal opportunities for success with e‐learning (Al‐Maskari et al., 2022; Zhao et al., 2021). Furthermore, as e‐learning technologies evolve, students must continually develop their computer skills to stay abreast of advancements and maintain a competitive edge in the job market (Bejaković & Mrnjavac, 2020).

The significance of student computer competency prompts further research questions:

Question 4: Is the relationship between virtual learning infrastructure and e‐learning outcomes explained by student computer competency?
Question 5: Is the relationship between student collaboration and e‐learning outcomes explained by student computer competency?
Question 6: Is the relationship between student access to electronic facilities and e‐learning outcomes explained by student computer proficiency?

### 2.5 E‐learning outcomes

The importance of e‐learning has increased in recent years (Attardi & Rogers, 2015); however, the opinions of nursing students on the effectiveness of e‐learning and associated learning outcomes vary. While some nursing students prefer traditional learning (Emami Sigaroudi et al., 2018), others perceive no difference between the two methods (Lahti et al., 2014). Some consider e‐learning to be more useful than traditional educational methods, believing that it can lead to desirable learning outcomes (Mosalanejad & Sobhanian, 2009). The importance of identifying factors that affect e‐learning outcomes cannot be overstated as it can help optimize the effectiveness of e‐learning and increase student satisfaction (Nortvig et al., 2018; Yekefallah et al., 2021). In addition to access to electronic facilities and student computer competency, other factors such as the design and delivery of course content, the quality of instructor feedback and support, and the level of student engagement can also significantly affect e‐learning outcomes (Cohen et al., 2013; Soong et al., 2001). Furthermore, cultural and social factors, including language barriers and varied expectations regarding educational experiences, can influence student perceptions of e‐learning (Pham & Tran, 2020). Consequently, educators and institutions must take these factors into account in the design and delivery of e‐learning programs, ensuring ongoing evaluation and adaptation of their approaches to optimize learning outcomes for students. In summary, numerous factors affect learning outcomes, and identifying these factors can help achieve desired educational outcomes. This prompts the seventh research question:

Question 7: What model is appropriate for achieving desired e‐learning outcomes, considering the roles of virtual education infrastructure, student collaboration, student access to electronic facilities, and student computer competency?

### 2.6 The current study

This study aims to investigate the relationships between virtual learning infrastructure, student collaboration, student access to electronic facilities, student computer competency, and e‐learning outcomes. While past research has underscored the significance of these factors in e‐learning (Lee et al., 2019; Nambiar, 2020; Sathyan et al., 2022; Tegowati et al., 2022) there exists a gap in comprehensive research examining their interrelationships and collective impact on e‐learning outcomes. Additionally, this study seeks to provide insight into the factors elucidating the relationships between these variables. Potential insights will aid in identifying factors that enhance e‐learning outcomes and inform strategies for educational institutions to bolster such outcomes.

## 3 METHOD

### 3.1 Study design

A cross‐sectional, correlational, descriptive design was employed to explore the mediating role of student computer competency in the relationship between virtual learning infrastructure, student collaboration, and access to electronic facilities with e‐learning outcomes among virtual student learners in Iran.

### 3.2 Participants

A prior sample size estimation was used to determine the minimum sample size required to avoid type I and type II errors. A minimum sample size of 382 was required based on five latent variables, 26 observed variables, a probability level less than 0.05, a power level of 0.8, and an effect size of 0.19 (Cohen et al., 2013). In total, 417 Iranian nursing students fulfilled the inclusion criteria and completed the questionnaire.

### 3.3 Measure

*Student computer competency* (5 items about students' experience using computer) and *Student collaboration* (5 items about students' participation in online learning discussions) scales were derived from Soong et al. (2001). In Pham and Tran (2020) study, the Cronbach's alpha for both subscales were 0.757. *Infrastructure* (4 items about efficient IT infrastructure) and *Accessibility* (6 items about access to the instructor, internet, and e‐learning websites) were from Volery and Lord (2000) study. The Cronbach's alpha for these subscales were 0.833 and 0.856, respectively (Pham & Tran, 2020). *Learning outcome (*6 items about effect of online courses on students' learning experience*)* was taken from the course valuing inventory (George & Mallery, 2020). Its Cronbach alpha is 0.874 in that study.

These five subscales are not validated among nursing students. The Participants rated each item on a 5‐point Likert scale, ranging from 1 (completely disagree) to 5 (completely agree). The Cronbach alpha values calculated in this study are reported in Table 3. Permission to use these scales has been obtained from Dr. Quoc Trung Pham.

### 3.4 Data collection

From August to October 2022, an online survey was conducted among Iranian university students using an online questionnaire platform. Participants, selected via a convenience sampling method, were Iranian nursing students who had e‐learning experience (synchronous). The online questionnaire was created using the Persian form platform (www.porsline.ir) and data was collected by sending the link to the questionnaire to students via Telegram, WhatsApp, email, or Persian social media.

### 3.5 Ethical considerations

Before engaging with the online questionnaire, students were provided with essential information, including research objectives and the number of items. Participants were informed that their participation was voluntary and that their confidential and anonymous information would be published in aggregate form. Respondents could only access items in the online survey after agreeing to participate by clicking the ‘Continue’ button, which also signified the completion of the online consent form. The protocol for this study was approved by the Ethics Committee of the Mazandaran University of Medical Sciences (Code of Ethics: IR.MAZUMS.REC.1401.260).

### 3.6 Data analysis

To examine the proposed mediation model, a two‐step process was used. First, descriptive statistics and correlation analysis were performed. Skewness and kurtosis were examined to check for normality, and scores less than 3 were considered acceptable (Kline, 2005). Next, a Pearson correlation analysis was performed to assess the relationship between infrastructure, access ability, student collaboration, student computer competency, and e‐learning outcomes. In the second part of the process, mediation models were used to test whether student computer competency mediated the association of infrastructure, access ability, and student collaboration with e‐learning outcomes. To test the proposed mediating model, the PROCESS macro v4.1 (Model 4) in SPSS v25 was used. The significance of the indirect effect of predictors on the outcome was evaluated using a bootstrap approach with 5000 resamples and 95% confidence intervals. All statistical analyses were performed using SPSS v25 on a Microsoft Windows platform (Mehrvarz et al., 2021; Preacher & Hayes, 2008).

## 4 RESULTS

The study sample comprised 417 students: 303 females (72.7%) and 114 males (27.3%), with a mean age of 28.84 years (SD = 9.51). The majority of respondents were single (*n* = 258, 61.9%). More than half of the nursing students were pursuing a bachelor's degree (58.5%).

Descriptive statistics indicated that skewness and kurtosis scores ranged from −0.59 to 2.54, suggesting an approximate normal distribution of the study variables (see Table 1). The correlation analysis revealed that infrastructure exhibited moderate‐to‐large correlations with access ability, student collaboration, student computer competency, and e‐learning outcomes. Significant and moderate correlations were found between access ability, student collaboration, student computer competency, and e‐learning outcomes. Finally, a significant, moderate correlation between student computer competency and e‐learning outcomes was observed (see Table 2).

**TABLE 1:** Descriptive statistics for the measures in the study.

	Min.	Max.	Mean	SD	Skewness	Kurtosis
Infrastructure	4.00	20.00	12.59	3.35	−0.24	0.28
Access ability	6.00	30.00	17.96	4.83	0.34	0.31
Students' collaboration	5.00	25.00	15.77	3.01	1.08	2.31
Students' computer competency	5.00	25.00	18.79	4.15	−0.51	0.17
E‐learning outcomes	6.00	30.00	20.28	4.76	−0.59	0.68

**TABLE 2:** Correlation results for the measures in the study.

	1.	2.	3.	4.	5.
1. Infrastructure	1	0.617	0.322	0.369	0.421
2. Access ability		1	0.290	0.395	0.441
3. Students' collaboration			1	0.256	0.295
4. Students' computer competency				1	0.330
5. E‐learning outcomes					1

*Note*: All correlations are significant at the 0.001 level (2‐tailed).

Three mediation models were used to examine whether student computer competency mediated the association between infrastructure, access ability, and student collaboration with e‐learning outcomes. Results revealed that infrastructure had a significant predictive effect on student computer competency (*β* = 0.30, *p* < 0.001) and e‐learning outcomes (*β* = 0.31, *p* < 0.001). E‐learning outcomes were also predicted by student computer competency (*β* = 0.16, *p* < 0.001). The results indicate that student computer competency mediated the association between infrastructure and e‐learning outcomes. Infrastructure accounted for 9% of the variance in student computer competency, and infrastructure and student computer competency accounted for 15% of the variance in e‐learning outcomes. Subsequent analyses revealed that access ability was a significant predictor of student computer competency (*β* = 0.28, *p* < 0.001) and e‐learning outcomes (*β* = 0.37, *p* < 0.001). E‐learning outcomes were predicted by student computer competency (*β* = 0.14, *p* = 0.002), and student computer competency mediated the association between access ability and e‐learning outcomes. Access ability accounted for 8% of the variance in student computer competency, and access ability and student computer competency accounted for 20% of the variance in e‐learning outcomes.

**FIGURE 1:**
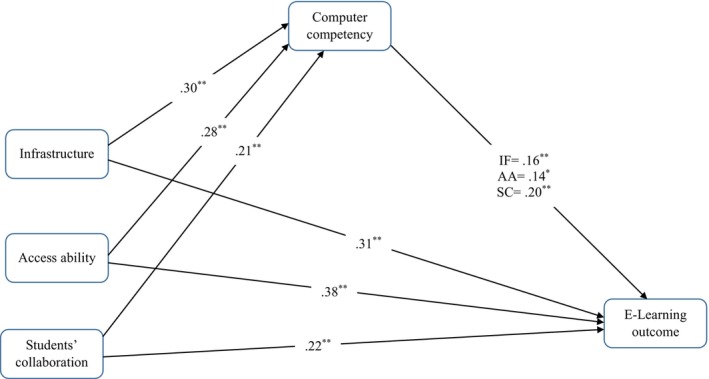
The mediation model. *Note*. **p < 0.001, *p < 0.01. AA, access ability; IF, infrastructure; SC, students' collaboration.

Results of the final mediation model indicated that student collaboration had a significant predictive effect on student computer competency (*β* = 0.21, *p* < 0.001) and e‐learning outcomes (*β* = 0.22, *p* < 0.001). E‐learning outcomes were also predicted by student computer competency (*β* = 0.20, *p* < 0.001), indicating that student computer competency mediated the association between student collaboration and e‐learning outcomes. Student collaboration accounted for 4% of the variance in student computer competency, and student collaboration and student computer competency accounted for 11% of the variance in e‐learning outcomes (see Table 3, Figure 1). Additionally, bootstrap results confirmed the proposed model, indicating significant indirect effects of infrastructure (*β* = 0.05, LLCI–ULCI = 0.01–0.08), access ability (*β* = 0.04, LLCI–ULCI = 0.01–0.07), and student collaboration (*β* = 0.04, LLCI–ULCI = 0.02–0.08) on student e‐learning outcomes through student computer competency (see Tables 4 and 5).

**TABLE 3:** Results of the measurement model assessment.

Construct	Factor loading	*α*	CR	AVE
**Items Infrastructure**				
1: I can use any PC at the university using the same account	0.50	0.72	0.71	0.40
2: I can use the computer labs for practicing	0.57			
3: The university internet connection is stable and secured	0.70			
4: Overall, the information technology infrastructure is efficient	0.71			
**Access ability**				
1: Easy on‐campus access to the internet	0.62	0.80	0.80	0.41
2: Did not experience problems while browsing	0.71			
3: Browsing speed was satisfactory	0.76			
4: I could interact with classmates through the web	0.57			
5: I could easily contact the instructor through e‐learning system	0.59			
6: Overall, the e‐learning website was easy to use	0.58			
**Students' collaboration**				
1: I do not read/participate in the discussion group on e‐learning	0.73	0.75	0.76	0.40
2: I only read messages in the discussion group on e‐learning	0.51			
3: I do read, as well as participate in the discussion group on e‐learning	0.71			
4: The instructor initiated most of the discussion on e‐learning	0.63			
5: The students initiated most of the discussion on e‐learning	0.56			
**Students' computer competency**				
1: I enjoy using personal computers	0.71	0.80	0.81	0.46
2: I use the personal computers for work and play	0.64			
3: I was comfortable with using the PC and software applications before I took up the e‐learning	0.65			
4: My previous experience in using the PC and software applications helped me in the e‐learning	0.72			
5: I am not intimidated by using the e‐learning‐based courses	0.65			
**Learning outcome**				
1: I believe that, this online course was a very valuable learning experience for me.	0.85	0.81	0.87	0.57
2: I believe that this online course was a constructive and definitely helpful learning experience	0.86			
3: Taking the online course made little difference for me	0.15			
4: In some ways I feel good about myself due to this online course	0.83			
5: Some of my values have been clarified due to this learning Experience	0.78			
6: This online course was useful in helping me develop new ways to achieve work tasks	0.81			

Abbreviations: α, Cronbach's alpha; AVE, average variance extracted; CR, composite reliability.

**TABLE 4:** Unstandardized estimates for the proposed mediation model.

Antecedent	Consequent			
	Coeff.	SE	*t*	*p*
	*M* (Students' computer competency)			
*X*_*1*_ (Infrastructure)	0.45	0.06	6.68	<0.001
Constant	13.03	0.89	14.62	<0.001
	*R* ^2^ = 0.14 *F* = 44.67; *p* < 0.001			
*X*_*2*_ (Access ability)	0.33	0.05	7.23	<0.001
Constant	12.70	0.87	14.58	<0.001
	*R* ^2^ = 0.16 *F* = 52.35; *p* < 0.001			
*X*_*3*_ (Students' collaboration)	0.35	0.08	4.47	<0.001
Constant	13.21	1.26	10.41	<0.001
	*R* ^2^ = 0.07 *F* = 19.97; *p* < 0.001			
	*Y* (E‐learning outcomes)			
*X*_*1*_ (Infrastructure)	0.49	0.08	6.09	<0.001
*M* (Students' computer competency)	0.23	0.06	3.57	<0.001
Constant	9.73	1.29	7.52	<0.001
	*R* ^2^ = 0.21 *F* = 38.15; *p* < 0.001			
*X*_*2*_ (Access ability)	0.36	0.05	6.45	<0.001
*M* (Students' computer competency)	0.21	0.06	3.24	<0.001
Constant	9.79	1.26	7.72	<0.001
	*R* ^2^ = 0.22 *F* = 40.69; *p* < 0.001			
*X*_*3*_ (Students' collaboration)	0.35	0.08	3.99	<0.001
*M* (Students' computer competency)	0.31	0.06	4.82	<0.001
Constant	8.79	1.62	5.41	<0.001
	*R* ^2^ = 0.16 *F* = 26.27; *p* < 0.001			

Abbreviations: Coeff, unstandardized coefficient; M, mediator variable; SE, standard error; X, independent variable; Y, outcome variable.

**TABLE 5:** Unstandardized estimates for the total, direct, and indirect effects.

Path	Effect	SE	LLCI	ULCI
Infrastructure→Computer Competency→E‐learning outcomes				
Total effect	0.60	0.07	0.45	0.75
Direct effect	0.49	0.08	0.33	0.65
Indirect effect	0.11	0.04	0.04	0.19
Access ability→Computer competency→E‐learning outcomes				
Total effect	0.43	0.05	0.33	0.54
Direct effect	0.36	0.05	0.25	0.47
Indirect effect	0.07	0.03	0.02	0.13
Students' collaboration→Computer competency→E‐learning outcomes				
Total effect	0.47	0.09	0.29	0.64
Direct effect	0.35	0.08	0.18	0.53
Indirect effect	0.11	0.04	0.04	0.19

*Note*: Number of bootstrap samples for percentile bootstrap confidence intervals: 5000.

## 5 DISCUSSION

With the growing prevalence of virtual learning environments (Mehrvarz et al., 2021), research has shown that virtual learning infrastructure, access ability, and student collaboration play a crucial role in shaping learning outcomes. This study aims to evaluate the mediating role of student computer competency in the relationship between virtual learning infrastructure, student collaboration, and access to electronic facilities, and their collective impact on e‐learning outcomes among online students in Iranian universities.

Overall, the study findings reveal a positive effect of virtual learning infrastructure (H1), student collaboration (H2), and access to electronic facilities (H3) on student e‐learning outcomes. From a technical perspective, and consistent with findings in the literature on the analysis of student learning outcomes, learning infrastructure guarantees e‐learning success (Alsabawy et al., 2013; Kumar et al., 2021). For this reason, students and teachers are increasingly reliant on technology for e‐learning, where a robust digital infrastructure underpins seamless access to facilitate effective online education (Tayyib et al., 2021). Furthermore, García (2016) revealed that a project‐based task that requires student collaboration in an online learning environment often leads to a more substantial knowledge gain, reflected in their academic performance (García, 2016). Similarly, Yew and Goh (2016) revealed that collaborative learning determines student learning outcomes (Yew & Goh, 2016). This aligns with Benbunan‐Fich and Arbaugh (2006), who asserted that online courses not only encourage students to take ownership of their own learning but also found that collaborative learning activities substantially increase opportunities for knowledge construction and performance improvement. Lastly, despite the contradictory results regarding the relationship between student's ability to access electronic resources and academic performance, our study indicates that proficiency with online learning technologies is very important for learning experiences. This is in line with existing literature, which indicates that the ability to access online learning facilities has a greater positive impact on student academic performance (Montenegro et al., 2016).

Results indicated that computer competence had a significant positive effect on student learning outcomes. In line with the results of Mehrvarz et al. (2021) and Castillo‐Merino & Serradell‐López (2014), student computer competence is the key determinant of student academic performance in online learning contexts. With higher levels of computer competency, students will gain a higher level of achievement in online learning settings. In addition, the results revealed that computer competence mediates the relationship between e‐learning infrastructure and learning outcomes (H4). For this reason, it is suggested that students possessing better computer competence are likely to utilize e‐learning infrastructures more effectively, thereby expanding their learning opportunities and overseeing their learning journey more effectively, resulting in improved learning outcomes. In this context, students' perceptions of their computer competence positively influence their learning skills and subsequently improve their performance in online learning scenarios (Castillo‐Merino & Serradell‐López, 2014). Thus, improved computer competency and access to e‐learning tools and resources will improve overall nursing student learning performance, extending beyond academic classes (Sharif‐Nia et al., 2023). Specifically, students who embody higher perceived computer competency will exhibit greater confidence in using electronic resources to facilitate their learning (Montenegro et al., 2016). Similarly, students who collaborate with peers in an online environment for knowledge construction are anticipated to exhibit greater confidence in their computer skills, likely leading to better learning outcomes. While empirical evidence on the mediation relationship is limited, it is reasonable to infer that nursing students with less computer anxiety will be more inclined to collaborate with peers to improve their learning performance (Papathanasiou et al., 2023). Thus, computer competency mediates the relationship between student collaboration and learning outcomes in an entirely web‐based learning environment.

### 5.1 Implications for nurse education

This study highlights the crucial role of computer competency in determining e‐learning outcomes, emphasizing the need for nursing students to possess the requisite computer skills to effectively engage in online learning. The findings also point to the substantial impact of virtual learning infrastructures and access to electronic facilities on nursing student computer competencies and e‐learning outcomes. Thus, prioritizing investment in high‐quality virtual learning environments and resources, and ensuring that students have access to the necessary technology and resources for e‐learning, should be paramount for anyone involved in e‐learning development, design, or implementation. A final implication of the study pertains to the significance of nursing student collaboration. The findings suggest that nursing student collaboration positively impacts computer competency, offering tangible benefits in an online learning context. The results emphasize the importance of fostering a collaborative learning environment that encourages students to cooperatively work and share knowledge.

### 5.2 Limitations and strengths

This study has several limitations that warrant caution in interpreting the results. First, the sample size, while statistically adequate for the analyses performed, is confined to Iranian nursing students, potentially limiting the generalizability to students in other countries or regions. Although the sample size was sufficient for the specific population of Iranian virtual students, it may not be expansive enough to extend the findings to a broader population of virtual students or to other populations and nations. Additionally, the study's cross‐sectional design presents another limitation, constraining our ability to establish cause‐and‐effect relationships between variables and assess the development of e‐learning outcomes over time. While the mean score of students' computer competency was moderate, there may be a recruitment bias due to the utilization of an online survey, which could marginalize students with lower competency levels. Future research, involving larger and more diverse samples across various populations or settings, and potentially employing a longitudinal design, is necessary to validate the findings of this study and to understand changes in e‐learning outcomes over a greater period of time.

The results of the study have important implications for the design and implementation of e‐learning programs, as well as for the development of technology‐augmented learning environments. Specifically, these findings underscore the need for interventions targeting the enhancement of peer‐to‐peer collaboration and computer competence, and ensuring students have access to the requisite tools and resources to improve their e‐learning experiences.

## 6 CONCLUSION

This study aimed to explore the mediating role of computer competency in the relationship between virtual learning infrastructure, student collaboration, and access to electronic facilities, with a focus on e‐learning outcomes among nursing students in Iran. The findings indicated that the virtual learning infrastructure, student collaboration, and access to electronic facilities significantly impact student computer competency, which subsequently influences e‐learning outcomes. These results shed light on the importance of student computer competency in enhancing the effectiveness of e‐learning and suggest that initiatives aimed at enhancing students' computer skills and ensuring access to virtual learning resources and facilities should be prioritized.

## AUTHOR CONTRIBUTIONS

F.KH, H.SH, and N.GH contributed to the study conception and design. Material preparation and data collection was performed by N.Gh, S.M., and E.H. Data analysis performed by H.SH and G.A. The first draft of the manuscript was written by K.A, L.M., L.SH, J.R., and P.R. All authors commented on previous versions of the manuscript. All authors read and approved the final manuscript.

## FUNDING INFORMATION

No funds, grants or other support was received.

## CONFLICT OF INTEREST STATEMENT

The authors report no conflicts of interest in this work.

## Data Availability

The data that support the findings of this study are available from the corresponding author upon reasonable request.
